# Deriving the priority weights from probabilistic linguistic preference relation with unknown probabilities

**DOI:** 10.1371/journal.pone.0208855

**Published:** 2018-12-10

**Authors:** Yongming Song

**Affiliations:** School of Business Administration, Shandong Technology and Business University, Yantai, China; Northeastern University, CHINA

## Abstract

Generally, the probabilistic linguistic term set (PLTS) provides more accurate descriptive properties than the hesitant fuzzy linguistic term set does. The probabilistic linguistic preference relation (PLPR), which is applied to deal with complex decision-making problems, can be constructed for PLTSs. However, it is difficult for decision makers to provide the probabilities of occurrence for PLPR. To deal with this problem, we propose a definition of expected consistency for PLPR and establish a probability computing model to derive probabilities of occurrence in PLPR with priority weights for alternatives. A consistency-improving iterative algorithm is presented to examine whether or not the PLPR is at an acceptable consistency. Moreover, the consistency-improving iterative algorithm should obtain the satisfaction consistency level for the unacceptable consistency PLPR. Finally, a real-world employment-city selection is used to demonstrate the effectiveness of the proposed method of deriving priority weights from PLPR.

## Introduction

In recent years, societal and technological trends have made decision-making environments more uncertain and complex. It is difficult for decision makers (DMs) to accurately evaluate alternatives using precise metrics. Accordingly, DMs have adopted a linguistic variable [1] to express their assessment results more flexibly and appropriately. A variety of linguistic models, including the fuzzy number-based model, the 2-tuple linguistic, and the virtual linguistic, have been introduced to deal with practical problems [2–4]. In real situations characterized by complexity and uncertainty, it is difficult for DMs to use single terms to indicate preference values. To address the difficulties of representing complex and uncertain situations with a single-term preference value, Torra [5] proposed the hesitant fuzzy set, which allowed DMs to simultaneously consider several hesitant values to produce an overall assessment. Catering to the cognitive characteristics of DMs, the hesitant fuzzy set incorporated an increased amount of influential data to avoid the loss of information. Furthermore, Rodriguez et al. [6] presented the hesitant fuzzy linguistic term set (HFLTS) to improve the richness of linguistic representation. In recent years, HFLTSs have obtained widespread attention from scholars [7–13]. All HFLTS terms provided by the decision maker (DM) have equal probabilities of occurrence. However, the probabilities of possible linguistic terms in HFLTS are not equal in some actual situations. For example, if a DM employs an HFLTS, {little good, good, very good}, to represent the control of a car, the DM may consider that the control level “little good” is the most desirable, whereas the control level “good” is more desirable than the control level “very good.” However, the control level “very good” is also possible. Thus, the HFLTS {little good, good, very good} cannot be used to represent this precise situation. Pang et al. [14] extended the HFLTS to probabilistic linguistic term set (PLTS), which allowed the DMs to provide multiple possible linguistic terms, considering simultaneous probabilities of occurrence for all possible linguistic terms. The PLTS, which enriches the expressive methods of linguistic information, provides more accurate descriptive properties than HFLTS. Therefore, it is better for the DMs to represent their preference information via PLTS. For the above example, the PLTS {little good (0.6), good (0.3), very good (0.1)} can be applied to represent the control of a car, because it contains more information than the HFLTS {little good, good, very good}. Recently, the mechanism and application of PLTS has aroused wide concern and has been a hot area of research in the decision-making fields [15–29].

Preference relations are pairwise comparisons of alternatives and are common representational tools for DMs. Based on PLTS, Zhang et al. [19] proposed the probabilistic linguistic preference relation (PLPR), which can be applied to solve multi-criteria decision-making problems. Generally, the PLPR decision matrix comprises the HFLTSs and their corresponding probabilities. The HFLTSs are easily given by DMs. However, it is difficult for DMs to provide probabilities for all linguistic terms in the HFLTSs. Intuitively, it is impossible or highly unlikely for DMs to provide exact probability values for all the linguistic terms. Therefore, establishing a probability computing model for the PLPR is a primary need. It is also the first objective of this paper: propose a probability computing model with unknown probabilities of PLPR.

It is well known that consistency is a significant issue widely considered in decision-making problems based on preference relations [30–33]. Consistency ensures DMs are logical and non-voluntary, making the result of decision-making credible. Therefore, PLPR consistency improvement is a critical issue. However, the consistency-improving algorithm of PLPR proposed by Zhang et al. [19] must adjust all elements in each iteration. This biases the original information of DMs. Therefore, to retain DMs’ original information as far as possible, the second objective of this paper is to establish a new method to improve the consistency of PLPR.

To solve the above problems, first, we introduce the expected consistency definition of the PLPR and establish its probability computing model to get the missing occurrence probabilities and priority weights of alternatives. Then, to improve the consistency level of PLPR, the expected consistency index (ECI) of the PLPR is developed. Moreover, for a PLPR with an unsatisfactory consistent level, an improving algorithm is proposed to obtain a PLPR with an acceptable expected consistency level.

The rest of this paper is organized as follows. Section 2 reviews some basic knowledge of linguistic information, linguistic preference relation (LPR), and PLPR. Section 3 presents a probability computation model of the PLPR based on the expected consistency. In Section 4, a consistency-improving algorithm is proposed. In Section 5, an employment-city selection problem is solved and a comparison analysis with other research is also presented. Section 6 gives the concluding remarks.

## Preliminaries

### Linguistic information

To facilitate linguistic assessment by DMs, the linguistic term set should be determined in advance. Generally, a linguistic term set has the following characteristics: an odd value with granularity; its membership functions are symmetrical and uniformly distributed; and the linguistic set midterm represents “indifference,” with the remainder of the linguistic terms symmetrically and uniformly placed on either side of it. Let *S* = {*s*_0_,*s*_1_,⋯,*s*_2*τ*_} be a linguistic term set with odd granularity. *s*_*i*_ represents the *i*^th^ linguistic term in *S*, and 2*τ*+1 is the granularity of the linguistic *S*. Moreover, the term set *S* should satisfy the following features [2]: a negation operator *Neg*(*s*_*i*_) = *s*_*j*_, such that *j* = 2*τ*−1−*i*; 2); an order *s*_*i*_≥*s*_*j*_, if *i*≥*j*.

To preserve all given information during the calculation process, a discrete linguistic term set *S* can be further expanded to the continuous form proposed by Xu [34], as follows:

$$\bar{S}=\left\{s_{\alpha }|\alpha \in \left[0,q\right]\right\},$$ (1)

where *q*>2*τ* is a large-enough positive integer. If *s*_*α*_∈*S*, the *s*_*α*_ is called an original term; otherwise, the *s*_*α*_ is a virtual term. Moreover, Xu [34] introduced operational laws for two linguistic terms, $s_{\alpha },s_{\beta }\in \bar{S}$ and *λ*,*λ*_1_,*λ*_2_∈[0,1], as follows:

$$s_{\alpha }⊕s_{\beta }=s_{\alpha +\beta };\;s_{\alpha }⊕s_{\beta }=s_{\beta }⊕s_{\alpha };\;\lambda s_{\alpha }=s_{\lambda \alpha };\;\lambda \left(s_{\alpha }⊕s_{\beta }\right)=\lambda s_{\alpha }⊕\lambda s_{\beta };\;\left(\lambda_{1}+\lambda_{2}\right)s_{\alpha }=\lambda_{1}s_{\alpha }⊕\lambda_{2}s_{\alpha }.$$

Furthermore, the subscript function *I*(⋅) and its inverse function *I*^−1^(⋅) are defined as follows:

$$I\left(s_{\alpha }\right)=\alpha ,\;I^{-1}\left(\alpha \right)=s_{\alpha }.$$ (2)

### Preference relations: LPR and PLPR

**Definition 1** ([35]). The *P* = (*p*_*ij*_)_*n*×*n*_ is called an LPR if the following conditions hold:

$$p_{ij}⊕p_{ji}=s_{2\tau },p_{ii}=s_{\tau },i,j\in N,$$ (3)

where *S* = {*s*_0_,*s*_1_,⋯,*s*_2*τ*_} is the given linguistic scale set.

Based on HFLTS, Pang et al. [14] proposed the PLTS as follows:

**Definition 2** ([14]). Let *S* = {*s*_0_,*s*_1_,⋯,*s*_2*τ*_} be a linguistic scale set. The definition of a PLTS is given as follows:

$$L\left(p\right)=\left\{L^{\left(k\right)}\left(p^{\left(k\right)}\right)|L^{\left(k\right)}\in S,p^{\left(k\right)}\geq 0,k=1,2,\cdots ,\#L\left(p\right),\sum_{k=1}^{\#L\left(p\right)}p^{\left(k\right)}\leq 1\right\},$$ (4)

where *L*^(*k*)^(*p*^(*k*)^) denotes the linguistic term, *L*^(*k*)^, associated with probability *p*^(*k*)^, whereas #*L*(*p*) suggests the number of all different linguistic terms in *L*(*p*).

Based on the PLTS, Zhang et al. [19] defined PLPR as follows:

**Definition 3**. A PLPR on set *X* is represented by a matrix *B* = (*L*_*ij*_(*p*))_*n*×*n*_⊂*X*×*X*, *i*,*j* = 1,2,⋯,*n*. Here *L*_*ij*_(*p*) = {*L*_*ij*,*k*_(*p*_*ij*,*k*_)|*k* = 1,2,⋯,#*L*_*ij*_} are PLTSs based on the given linguistic scale set, *S* = {*s*_0_,*s*_1_,⋯,*s*_2*τ*_}, where $p_{ij,k}\geq 0,\sum_{k=1}^{\#L_{ij}\left(p\right)}p_{ij,k}=1$, and #*L*_*ij*_(*p*) represents the number of linguistic terms in *L*_*ij*_(*p*). *L*_*ij*_(*p*) is expressed as the preference degrees of alternative *x*_*i*_ over *x*_*j*_ and satisfies the following conditions:

$$p_{ij,k}=p_{ji,k},L_{ij,k}+L_{ji,k}=s_{2\tau },L_{ii}\left(p\right)=\left\{s_{\tau }\left(1\right)\right\},\#L_{ij}=\#L_{ji},$$ (5)

$$L_{ij,k}<L_{ij,k+1},L_{ji,k}>L_{ji,k+1},\;for\;i>j,$$ (6)

where *L*_*ij*,*k*_ and *p*_*ij*,*k*_ are the *k*^th^ linguistic term and the occurrence probability of the *k*^th^ linguistic term in *L*_*ij*_(*p*), respectively.

## Probability computation model of the PLPR based on expected consistency

Zhang et al. [19] defined PLPR consistency using a normalization process. However, the normalization process-based method had two features. It extended all PLTSs to the length of the PLTS with the biggest number of elements, and it used the ordered PLTSs to judge the consistency of associated PLPR. The normalization process-based method restricted the consistency of ordered PLTSs. To address these limitations, this paper defines a new expected consistency for PLPR. In contrast to the previous consistency concepts, this new concept does not add values to be provided by the DMs.

To calculate the probability of a PLPR, we present a probability computation model in which the definition of the expected consistency of a PLPR is based on the additive consistency of LPR [36]. We give preliminary knowledge as follows:

**Definition 4**. Given a PLTS *L*(*p*) = {*L*_*k*_(*p*_*k*_)|*k* = 1,2,⋯,#*L*(*p*)}, then its expected value can be defined as

$$E\left(L\left(p\right)\right)=\bar{e}=\sum_{k=1}^{\#L\left(p\right)}I\left(L_{k}\right)\cdot p_{k}$$ (7)

where #*L*(*p*) is the number of possible elements in *L*(*p*).

**Definition 5 (**[36]). An LPR, *A* = (*a*_*ij*_)_*n*×*n*_, is an additive consistent LPR if it satisfies the following conditions:

$$I\left(a_{ij}\right)=\tau +2\tau \left(\omega_{i}-\omega_{j}\right),$$ (8)

where *S* = {*s*_0_,*s*_1_,⋯,*s*_2*τ*_} is given a linguistic scale set and *ω* = {*ω*_1_,*ω*_2_,⋯,*ω*_*n*_}^*T*^ is the priority weights of *A*, meeting $\sum_{i=1}^{n}\omega_{i}=1$ and *ω*_*i*_≥0,*i* = 1,2,⋯,*n*.

Based on the additive consistency of LPR, the expected consistency of PLPR is presented as follows:

**Definition 6.** Given a set of alternatives, *X* = {*x*_1_,*x*_2_,⋯,*x*_*n*_}, *H* = (*L*_*ij*_(*p*))_*n*×*n*_⊂*X*×*X*, *i*,*j* = 1,2,⋯,*n* is a PLPR for *X*, where *L*_*ij*_(*p*) = {*L*_*ij*,*k*_(*p*_*ij*,*k*_)|*k* = 1,2,⋯,#*L*_*ij*_} is a PLTS expressed as the preference degrees of the alternative *x*_*i*_ over *x*_*j*_. Then, *H* is the expectant consistency if ${\bar{e}}_{ij}+{\bar{e}}_{jk}+{\bar{e}}_{ki}={\bar{e}}_{ik}+{\bar{e}}_{kj}+{\bar{e}}_{ji},\;\forall i,j,k\in N$, as expressed below.

$$\bar{e_{ij}}=\sum_{k=1}^{\#L_{ij}}I\left(L_{ij,k}\right)\cdot p_{ij,k}=\tau +2\tau \left(\omega_{i}-\omega_{j}\right),\;i,j=1,2,\cdots ,n,$$ (9)

where *k* = 1,2,⋯,#*L*_*ij*_, and #*L*_*ij*_ is the number of possible linguistic terms in *L*_*ij*_.

Moreover, based on the expectant consistency of PLPR, the probability computing model can be developed with the following mathematical programming:

$$\begin{array}{l} \min\;\varepsilon_{ij}=\left|2\tau \left(\omega_{i}-\omega_{j}\right)+\tau -\bar{e_{ij}}\right|\\ \;=\left|2\tau \left(\omega_{i}-\omega_{j}\right)+\tau -\sum_{k=1}^{\#L_{ij}}I\left(L_{ij,k}\right)\cdot p_{ij,k}\right|\\ \;s.t.\;\left\{ \begin{array}{l} \sum_{i=1}^{n}\omega_{i}=1,\omega_{i}\geq 0\\ i,j=1,2,\cdots ,n \end{array}\right. \end{array}$$ (10)

To simplify Eq (10), we present Theorem 1:

**Theorem 1.** For Eq (10), the following relationship is established:

$$\left|2\tau \left(\omega_{j}-\omega_{i}\right)+\tau -\sum_{k=1}^{\#L_{ij}}I\left(L_{ji,k}\right)\cdot p_{ji,k}\right|=\left|2\tau \left(\omega_{i}-\omega_{j}\right)+\tau -\sum_{k=1}^{\#L_{ij}}I\left(L_{ij,k}\right)\cdot p_{ij,k}\right|$$ (11)

**Poof.** Based on the properties of the PLPR, we have

$$I\left(L_{ji,k}\right)=2\tau -I\left(L_{ij,k}\right),\;p_{ji,k}=p_{ij,k}.$$

Then,

$$\begin{array}{l} \left|2\tau \left(\omega_{j}-\omega_{i}\right)+\tau -\sum_{k=1}^{\#L_{ij}}I\left(L_{ji,k}\right)\cdot p_{ji,k}\right|=\left|2\tau \left(\omega_{j}-\omega_{i}\right)+\tau -\sum_{k=1}^{\#L_{ij}}\left(2\tau -I\left(L_{ij,k}\right)\right)\cdot p_{ji,k}\right|\\ \;=\left|2\tau \left(\omega_{j}-\omega_{i}\right)+\tau -\sum_{k=1}^{\#L_{ij}}\left(2\tau -I\left(L_{ij,k}\right)\right)\cdot p_{ij,k}\right|\\ \;=\left|2\tau \left(\omega_{j}-\omega_{i}\right)-\tau +\sum_{k=1}^{\#L_{ij}}I\left(L_{ij,k}\right)\cdot p_{ij,k}\right|\\ \;=\left|2\tau \left(\omega_{i}-\omega_{j}\right)+\tau -\sum_{k=1}^{\#L_{ij}}I\left(L_{ij,k}\right)\cdot p_{ij,k}\right| \end{array}.$$

This completes the proof of Theorem 1.

According to Theorem 1, Eq (10) is simplified to the following:

$$\begin{array}{l} \min\;\varepsilon_{ij}=\left|2\tau \left(\omega_{i}-\omega_{j}\right)+\tau -\bar{e_{ij}}\right|\\ \;=\left|2\tau \left(\omega_{i}-\omega_{j}\right)+\tau -\sum_{k=1}^{\#L_{ij}}I\left(L_{ij,k}\right)\cdot p_{ij,k}\right|\\ \;s.t.\;\left\{ \begin{array}{l} \sum_{i=1}^{n}\omega_{i}=1,\omega_{i}\geq 0\\ i,j=1,2,\cdots ,n,\;i<j \end{array}\right. \end{array}$$ (12)

Moreover, Eq (12) can be transformed into the following mathematical programming:

$$\begin{array}{l} \min\;f=\sum_{i=1}^{n-1}\sum_{j=2,j>i}^{n}\left(t_{ij}d_{ij}^{+}+m_{ij}d_{ij}^{-}\right)\\ \;s.t.\;\left\{ \begin{array}{l} 2\tau \left(\omega_{i}-\omega_{j}\right)+\tau -\sum_{k=1}^{\#L_{ij}}I\left(L_{ij,k}\right)\cdot p_{ij,k}-t_{ij}d_{ij}^{+}+m_{ij}d_{ij}^{-}=0\\ \;d_{ij}^{+},d_{ij}^{-}\geq 0\\ \;\sum_{i=1}^{n}\omega_{i}=1,\omega_{i}\geq 0\\ \;i,j=1,2,\cdots ,n,\;i<j \end{array}\right. \end{array}$$ (13)

where $d_{ij}^{+}$ and $d_{ij}^{-}$ are the positive and negative deviations with respect to the goal, *ε*_*ij*_, respectively. *t*_*ij*_ and *m*_*ij*_ are the weights corresponding to $d_{ij}^{+}$ and $d_{ij}^{-}$, respectively. Here, the mathematical programming, Eq (13), can be called the probability computing model and can be used to compute the occurrence probabilities in the PLPR.

Generally, we assume that all goals, *ε*_*ij*_ (*i*,*j* = 1,2,⋯,*n*,*i*<*j*), are fair, and then *t*_*ij*_ = *m*_*ij*_ = 1 (*i*,*j* = 1,2,⋯,*n*,*i*<*j*). Hence, Eq (13) can be translated into the following:

$$\begin{array}{l} \min\;f=\sum_{i=1}^{n-1}\sum_{j=2,j>i}^{n}\left(d_{ij}^{+}+d_{ij}^{-}\right)\\ \;s.t.\;\left\{ \begin{array}{l} 2\tau \left(\omega_{i}-\omega_{j}\right)+\tau -\sum_{k=1}^{\#L_{ij}}I\left(L_{ij,k}\right)\cdot p_{ij,k}-d_{ij}^{+}+d_{ij}^{-}=0\\ \;d_{ij}^{+},d_{ij}^{-}\geq 0\\ \;\sum_{i=1}^{n}\omega_{i}=1,\omega_{i}\geq 0\\ \;i,j=1,2,\cdots ,n,\;i<j \end{array}\right. \end{array}$$ (14)

Probabilities of occurrence in the PLTS can be obtained by applying the above computing model, Eq (14). Moreover, it is noteworthy that the priority weights can also be derived by Eq (14). In the following, an example is solved.

**Example 1.** Given a linguistic term set, *S* = {*s*_0_,*s*_1_,⋯,*s*_8_}, *H* is a PLPR with unknown probabilities, as shown below.

$$H=\left[\begin{array}{ccc} s_{4} & \left\{s_{2}\left(p_{12,1}\right),s_{3}\left(p_{12,2}\right)\right\} & \left\{s_{1}\left(p_{13,1}\right),s_{4}\left(p_{13,2}\right)\right\}\\ \left\{s_{6}\left(p_{21,1}\right),s_{5}\left(p_{21,2}\right)\right\} & s_{4} & \left\{s_{5}\left(p_{23,1}\right),s_{6}\left(p_{23,2}\right)\right\}\\ \left\{s_{7}\left(p_{31,1}\right),s_{4}\left(p_{31,2}\right)\right\} & \left\{s_{3}\left(p_{32,1}\right),s_{2}\left(p_{32,2}\right)\right\} & s_{4} \end{array}\right].$$

Based on Eq (14), we obtain the following goal programming:

$$\begin{array}{l} \min\;f=d_{12}^{+}+d_{12}^{-}+d_{13}^{+}+d_{13}^{-}+d_{23}^{+}+d_{23}^{-}\\ s.t.\left\{ \begin{array}{l} 8\omega_{1}-8\omega_{2}+4-\left(2p_{12,1}+3p_{12,2}\right)-d_{12}^{+}+d_{12}^{-}=0\\ 8\omega_{1}-8\omega_{3}+4-\left(p_{13,1}+4p_{13,2}\right)-d_{13}^{+}+d_{13}^{-}=0\\ 8\omega_{2}-8\omega_{3}+4-\left(5p_{23,1}+6p_{23,2}\right)-d_{23}^{+}+d_{23}^{-}=0\\ p_{12,1}+p_{12,2}=1,p_{13,1}+p_{13,2}=1,p_{23,1}+p_{23,2}=1\\ \omega_{1}+\omega_{2}+\omega_{3}=1\\ p_{12,1},p_{12,2},p_{13,1},p_{13,2},p_{23,1},p_{23,2}\geq 0\\ d_{12}^{+},d_{12}^{-},d_{13}^{+},d_{13}^{-},d_{23}^{+},d_{23}^{-}\geq 0\\ \omega_{1},\omega_{2},\omega_{3}\geq 0 \end{array}\right. \end{array}$$

Via MATLAB, the optimal solutions are obtained as follows:

$$\begin{array}{l} \omega ={\left(0.243,0.4582,0.2988\right)}^{T};\;p_{12,1}=0.721,p_{12,2}=0.279,p_{13,1}=0.1487,p_{13,2}=0.8531,\\ p_{23,1}=0.725,p_{23,2}=0.275;d_{12}^{+}=d_{12}^{-}=d_{13}^{+}=d_{13}^{-}=d_{23}^{+}=d_{23}^{-}=0. \end{array}$$

Thus, the complete PLPR is then obtained as follows:

$$H_{1}=\left[\begin{array}{ccc} s_{4}\left(1\right) & \left\{s_{2}\left(0.721\right),s_{3}\left(0.279\right)\right\} & \left\{s_{1}\left(0.1487\right),s_{4}\left(0.8513\right)\right\}\\ \left\{s_{6}\left(0.721\right),s_{5}\left(0.279\right)\right\} & s_{4}\left(1\right) & \left\{s_{5}\left(0.725\right),s_{6}\left(0.275\right)\right\}\\ \left\{s_{7}\left(0.1487\right),s_{4}\left(0.8513\right)\right\} & \left\{s_{3}\left(0.725\right),s_{2}\left(0.275\right)\right\} & s_{4}\left(1\right) \end{array}\right].$$

According to *ω* = (0.243,0.4582,0.2988)^*T*^, we derive the ranking of alternatives, *x*_2_≻*x*_3_≻*x*_1_.

Furthermore, the PLPR is at the expected consistency if *f* = 0 in Eq (14). However, this situation rarely occurs (i.e., the PLPR may be inconsistent). To deal with this problem, a consistency-improving algorithm of PLPR is presented next.

## Consistency improvement of the PLPR

In this section, to examine the expected consistency level of the PLPR, the ECI of PLPR is defined, and a consistency-improving algorithm is constructed to improve the expectant consistency level by adjusting certain elements of the PLPR. When the ECI of the PLPR achieves or exceeds the presupposed satisfactory level, $\bar{ECI}$, the consistency-improving algorithm is finished. Otherwise, the probability computing model can be applied again to get the probabilities of all linguistic terms in the acceptably consistent PLPR. Next, a numerical example is provided to show the feasibility and practicality of the consistency-improving algorithm.

**Definition 7.** Suppose *H* = (*L*_*ij*_(*p*))_*n*×*n*_ (*i*,*j* = 1,2,⋯,*n*) is a PLPR based on the given linguistic scale set, *S* = {*s*_0_,*s*_1_,⋯,*s*_2*τ*_}, where *L*_*ij*_(*p*) = {*L*_*ij*,*k*_(*p*_*ij*,*k*_)|*k* = 1,2,⋯,#*L*_*ij*_}. #*L*_*ij*_ is the number of possible linguistic terms in *L*_*ij*_. Furthermore, $d_{ij}^{+}$ and $d_{ij}^{-}$ are the positive and negative deviations with respect to goal *ε*_*ij*_ in Eq (14), respectively. Then, the ECI of the PLPR is defined as

$$ECI\left(H\right)=1-\frac{\sum_{i=1}^{n-1}\sum_{j=2,j>i}^{n}\left(d_{ij}^{+}+d_{ij}^{-}\right)}{\tau n\left(n-1\right)}.$$ (15)

If *ECI*(*H*) = 1, the PLPR meets the expected consistency perfectly. Otherwise, the expected consistency degree is reduced. In a real decision-making environment, it is difficult to get completely consistent PLPRs for DMs. According to practical decision problems, the threshold value of consistency-level $\bar{ECI}$ is determined in advance. Typically, the $\bar{ECI}$ may be set to 0.99, 0.98, and 0.95, corresponding to the expected consistent level of 99%, 98%, and 95%, respectively. To obtain credible decision-making results, the expected consistency level must achieve or surpass the threshold value of consistency-level $\bar{ECI}$. Otherwise, we should improve the expected consistency level.

**Fig 1:**
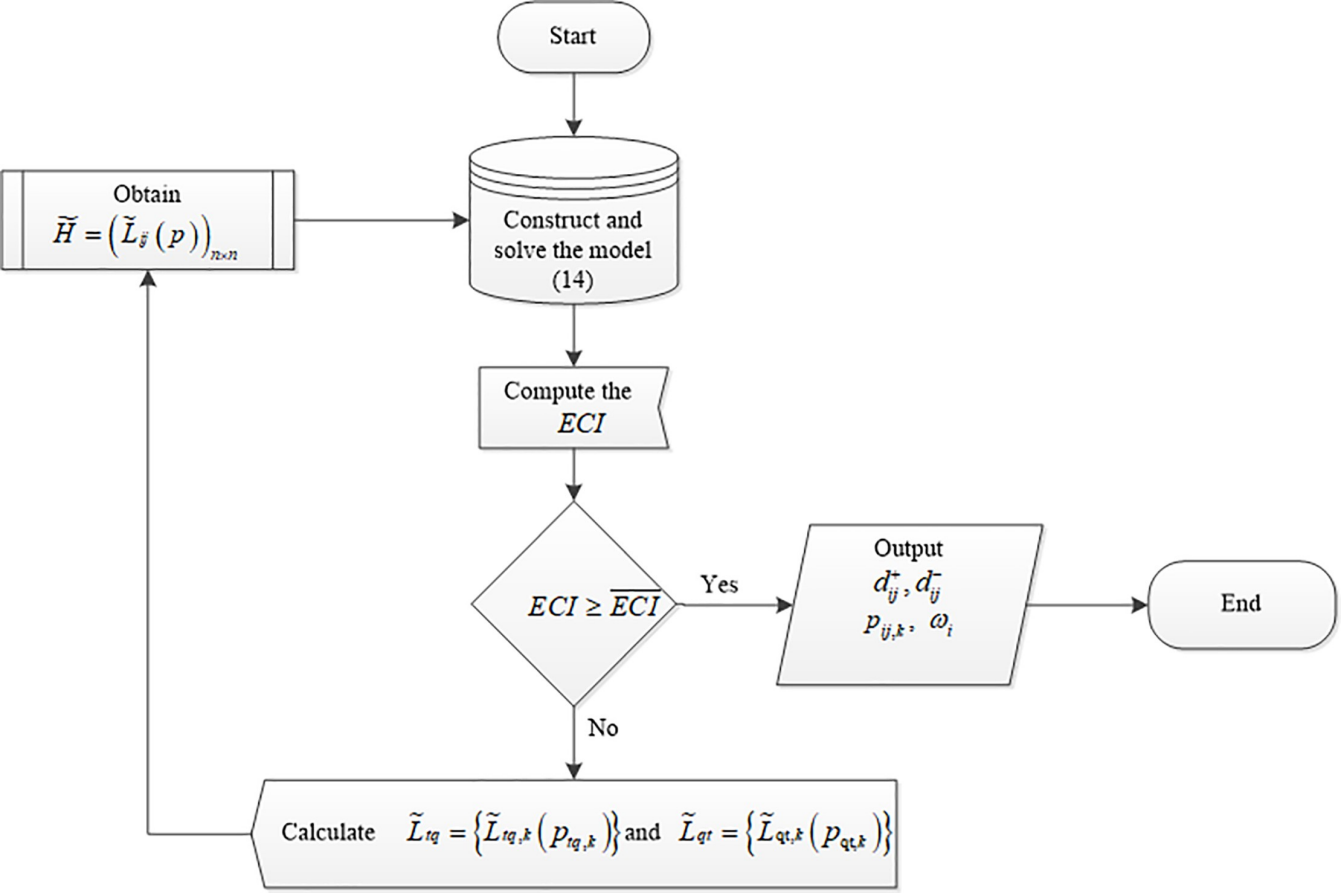
Consistency-improving process based on an iterative algorithm.

In what follows, we establish a consistency-improving iterative algorithm whose basic architectural scheme is shown in Fig 1, and we give the specific implementation steps.

### Consistency-improving iterative algorithm

**Step 1.** Calculate the positive deviations, $d_{ij}^{+}$, negative deviations, $d_{ij}^{-}$, occurrence probabilities, $p_{ij}^{\left(k\right)}$, and priority weights, *ω* = {*ω*_1_,*ω*_2_,⋯,*ω*_*n*_}^*T*^ of PLPR *H* = (*L*_*ij*_(*p*))_*n*×*n*_ (*i*,*j* = 1,2,⋯,*n*) by using the proposed probability computing Eq (14).

**Step 2.** Set the threshold value of the consistency-level $\bar{ECI}$ and calculate *ECI*(*H*) with $ECI\left(H\right)=1-\frac{\sum_{i=1}^{n-1}\sum_{j=2,j>i}^{n}\left(d_{ij}^{+}+d_{ij}^{-}\right)}{\tau n\left(n-1\right)}$. If $ECI\left(H\right)\geq \bar{ECI}$, then Output *p*_*ij*,*k*_ and *ω*_*i*_ (*i*,*j* = 1,2,⋯,*n*; *k* = 1,2,⋯,#*L*_*ij*_); otherwise, proceed to next step.

**Step 3.** Compute the maximum deviation, *d*^max^, using Eq (16).

$$d^{\max}=\max\left\{d_{ij}^{+},d_{ij}^{-}|i=1,2,\cdots ,n-1;j=1,2,\cdots ,n;j>i\right\}$$ (16)

**Step 4.** Based on the obtained *d*^max^, we are left with two situations, described in detail as follows:

1. If $d^{\max}=d_{tq}^{+}$ where *t* = 1,2,⋯,*n*−1,*q* = 2,3,⋯,*n*, and *q*>*t*, then we calculate the corrective elements, ${\tilde{L}}_{tq,k}$, according to ${\tilde{L}}_{tq,k}=I^{-1}\left(I\left(L_{tq,k}\right)+d_{tq}^{+}\right)$, where *k* = 1,2,⋯,#*L*_*tq*_.
2. If $d^{\max}=d_{tq}^{-}$, where *t* = 1,2,⋯,*n*−1,*q* = 2,3,⋯,*n*, and *q*>*t*, then we calculate the corrective elements, ${\tilde{L}}_{tq,k}$, according to ${\tilde{L}}_{tq,k}=I^{-1}\left(I\left(L_{tq,k}\right)-d_{tq}^{-}\right)$, where *k* = 1,2,⋯,#*L*_*tq*_.

Based on the above analysis, we can get the corrective PLPR, $\tilde{H}={\left({\tilde{L}}_{ij}\left(p\right)\right)}_{n\times n}$, based on the corrective elements, ${\tilde{L}}_{tq,k}$. The iterative process returns to **Step 1**.

If the expectant consistency level of the corrective PLPR has not met the requirement, then, the consistency-improving algorithm should be reused until the consistency level is acceptable. Example 2 further provides concrete details of the consistency-improving algorithm.

**Example 2.** Given a PLPR, *H*, we get

$$H=\left[\begin{array}{ccc} \left\{s_{4}\left(1\right)\right\} & \left\{s_{2}\left(p_{12,1}\right),s_{3}\left(p_{12,2}\right)\right\} & \left\{s_{5}\left(p_{13,1}\right),s_{6}\left(p_{13,2}\right)\right\}\\ \left\{s_{6}\left(p_{21,1}\right),s_{5}\left(p_{21,2}\right)\right\} & \left\{s_{4}\left(1\right)\right\} & \left\{s_{4}\left(1\right)\right\}\\ \left\{s_{3}\left(p_{31,1}\right),s_{2}\left(p_{31,2}\right)\right\} & \left\{s_{4}\left(1\right)\right\} & \left\{s_{4}\left(1\right)\right\} \end{array}\right].$$

According to Eq (14), we establish the following goal programming:

$$\begin{array}{l} \min\;f=d_{12}^{+}+d_{12}^{-}+d_{13}^{+}+d_{13}^{-}+d_{23}^{+}+d_{23}^{-}\\ s.t.\left\{ \begin{array}{l} 8\omega_{1}-8\omega_{2}+4-\left(2p_{12,1}+3p_{12,2}\right)-d_{12}^{+}+d_{12}^{-}=0\\ 8\omega_{1}-8\omega_{3}+4-\left(5p_{13,1}+6p_{13,2}\right)-d_{13}^{+}+d_{13}^{-}=0\\ 8\omega_{2}-8\omega_{3}-d_{23}^{+}+d_{23}^{-}=0\\ p_{12,1}+p_{12,2}=1,p_{13,1}+p_{13,2}=1\\ \omega_{1}+\omega_{2}+\omega_{3}=1\\ p_{12,1},p_{12,2},p_{13,1},p_{13,2}\geq 0\\ d_{12}^{+},d_{12}^{-},d_{13}^{+},d_{13}^{-}\geq 0\\ \omega_{1},\omega_{2},\omega_{3}\geq 0 \end{array}\right. \end{array}.$$

Via MATLAB, we obtain the optimal solution as follows:

$$\begin{array}{l} \omega ={\left(0.3333,0.3749,0.2918\right)}^{T};\;p_{12,1}=0,p_{12,2}=1,p_{13,1}=1,p_{13,2}=0;\\ d_{12}^{+}=0.6671,d_{12}^{-}=0,d_{13}^{+}=0,d_{13}^{-}=0.6675,d_{23}^{+}=0.6654,d_{23}^{-}=0. \end{array}$$

Therefore, *ECI*(*H*) = 0.9167 is obtained from Eq (15). In this example, we set $\bar{ECI}=0.95$. Because *ECI*(*H*)<0.95, we must carry out the consistency-improving iterative algorithm as follows:

According to Eq (16), we obtain $d^{\max}=d_{13}^{-}=0.6675$. Then, according to ${\tilde{L}}_{13,k}=I^{-1}\left(I\left(L_{13,k}\right)-d_{13}^{-}\right)\left(k=1,2\right)$, we get optimal elements ${\tilde{L}}_{13,1}=s_{4.33}$ and ${\tilde{L}}_{13,2}=s_{5.33}$. The corrective PLTS is ${\tilde{L}}_{13}=\left\{s_{4.33}\left(p_{13,1}\right),s_{5.33}\left(p_{13,2}\right)\right\}$, Thus, another corrective PLTS is ${\tilde{L}}_{31}=\left\{s_{3.67}\left(p_{31,1}\right),s_{2.67}\left(p_{31,2}\right)\right\}$ and the corrective PLPR is as follows:

$$\tilde{H}=\left[\begin{array}{ccc} \left\{s_{4}\left(1\right)\right\} & \left\{s_{2}\left(p_{12,1}\right),s_{3}\left(p_{12,2}\right)\right\} & \left\{s_{4.33}\left(p_{13,1}\right),s_{5.33}\left(p_{13,2}\right)\right\}\\ \left\{s_{6}\left(p_{21,1}\right),s_{5}\left(p_{21,2}\right)\right\} & \left\{s_{4}\left(1\right)\right\} & \left\{s_{4}\left(1\right)\right\}\\ \left\{s_{3.67}\left(p_{31,1}\right),s_{2.67}\left(p_{31,2}\right)\right\} & \left\{s_{4}\left(1\right)\right\} & \left\{s_{4}\left(1\right)\right\} \end{array}\right].$$

By means of Eq (14), we obtain the optimal solution as follows:

$$\begin{array}{l} \bar{\omega }={\left(0.3063,0.3753,0.3184\right)}^{T},\;p_{12,1}=0,p_{12,2}=1,p_{13,1}=1,p_{13,2}=0,\\ d_{12}^{+}=0.4486,d_{12}^{-}=0,d_{13}^{+}=0,d_{13}^{-}=0.4268,d_{23}^{+}=0.4546,d_{23}^{-}=0. \end{array}$$

Therefore, we obtain $ECI\left(\tilde{H}\right)=0.9446$ based on Eq (15). Because $ECI\left(\tilde{H}\right)<0.95$, we must keep improving the expected consistency of $\tilde{H}$ with the consistency-improving iterative algorithm as follows:

According to Eq (16), we obtain $d^{\max}=d_{23}^{+}=0.4546$. Thus, according to ${\tilde{L}}_{23}=I^{-1}\left(I\left(L_{23}\right)+d_{23}^{+}\right)$, we get the optimal element, ${\tilde{L}}_{23}=s_{4.45}$, and the corrected PLTS is ${\tilde{L}}_{23}=\left\{s_{4.45}\left(1\right)\right\}$. Thus, another corrective PLTS is ${\tilde{L}}_{32}=\left\{s_{3.55}\left(1\right)\right\}$, and the corrective PLPR is as follows:

$$\tilde{\tilde{H}}=\left[\begin{array}{ccc} \left\{s_{4}\left(1\right)\right\} & \left\{s_{2}\left(p_{12,1}\right),s_{3}\left(p_{12,2}\right)\right\} & \left\{s_{4.33}\left(p_{13,1}\right),s_{5.33}\left(p_{13,2}\right)\right\}\\ \left\{s_{6}\left(p_{21,1}\right),s_{5}\left(p_{21,2}\right)\right\} & \left\{s_{4}\left(1\right)\right\} & \left\{s_{4.45}\left(1\right)\right\}\\ \left\{s_{3.67}\left(p_{31,1}\right),s_{2.67}\left(p_{31,2}\right)\right\} & \left\{s_{3.55}\left(1\right)\right\} & \left\{s_{4}\left(1\right)\right\} \end{array}\right].$$

By means of Eq (14), we have the following optimal solution:

$$\begin{array}{l} \bar{\bar{\omega }}={\left(0.307,0.3947,0.2983\right)}^{T},\;p_{12,1}=0,p_{12,2}=1,p_{13,1}=1,p_{13,2}=0,\\ d_{12}^{+}=0.2985,d_{12}^{-}=0,d_{13}^{+}=0,d_{13}^{-}=0.2604,d_{23}^{+}=0.3211,d_{23}^{-}=0. \end{array}$$

Therefore, we obtain $ECI\left(\tilde{\tilde{H}}\right)=0.9633>0.95$ based on Eq (15). Then, PLPR $\tilde{\tilde{H}}$ is at the expected consistency with a confidence level of 95%. Therefore, the consistency-improving iterative algorithm is finished. Based on $\bar{\bar{\omega }}={\left(0.307,0.3947,0.2983\right)}^{T}$, we obtain the ranking of alternatives: *x*_2_≻*x*_1_≻*x*_3_.

The ranking of alternatives has been changed with the consistency-improving iterative algorithm. Table 1 shows the comparison of rankings before and after consistency improvement. It shows that the ranking between *x*_1_ and *x*_3_ is changed before and after consistency improvement. The ranking, *x*_1_≻*x*_3_, is more credible, because the PLPR of acceptable consistency level ensured by the DMs is logical and non-voluntary. Therefore, the consistency-improvement process plays an important role in decision results.

**Table 1:** The ranking of alternatives.

Consistency improving	Ranking of alternative
**Before**	*x*_2_≻*x*_3_≻*x*_1_
**After**	*x*_2_≻*x*_1_≻*x*_3_

## Calculating-examples analysis and contrastive analysis

### Employment-city selection problem

Because the reforms and increased openness of China, beginning in the 1980s, large-scale population migration and rapid urbanization have attracted domestic and international scholarly interest [37–39]. Selection of employment location is the key determinant of worker movement [40–42]. The influences on worker employment location selection can be grouped into internal and external factors. The internal factors are age, gender, and education, and the main external influences include social networks, the economy, and the environment [41]. Some researchers pointed out that, in addition to personal characteristics of migrant workers that might influence their employment location selection, the development level of the manufacturing industry, the level of economic development, the salary level, and the working environment also directly affected the choice of employment location [42–44]. However, prior studies primarily used qualitative description rather than quantitative methods to study employment location. In what follows, we study the employment-city selection problem using the proposed method from a quantitative perspective.

The employment-city selection of a graduate plays a crucial role in discriminating candidates for an enterprise and has a great impact on allocation of regional human resources and regional socio-economic operations and development. In China, with the deepening reform of the economic and educational systems, it is increasingly prominent for the employment structure of graduates to lose balance. The Ministry of Education and relevant departments have implemented policies and have conducted related research projects, hoping to explore a new path by retaining and attracting talent to achieve coordinated development of the regional economy. A graduating PhD from China is assumed to select from the following four cities to take up an occupation: *x*_*i*_(*i* = 1,2,3,4):*x*_1_: Qing Dao; *x*_2_: Zheng Zhou; *x*_3_: Shi Jia Zhuang; *x*_4_: Da Lian. For a graduating PhD, job availability, wages, work intensity and environmental conditions in the employment location directly influence the decision regarding where to migrate. Based on this, the graduating PhD makes a preference decision using four criteria to compare the cities (i.e., air cleanliness (*c*_1_), the level of urban development (*c*_2_), salary and mean consumption ratio (*c*_3_), and house prices level (*c*_4_).

The graduating PhD first provides her/his preference information with *x*_*i*_ over *x*_*j*_(*i*,*j* = 1,2,3,4) using PLTS, which represents the preferences of the graduating PhD over each pair of cities (*x*_*i*_,*x*_*j*_), based on the criterion, *c*_*l*_(*l* = 1,2,3,4). The corresponding PLPRs are shown in Tables 2–5. Here, the graduating PhD applies a set of nine linguistic terms:

$$S=\left\{\begin{array}{l} s_{0}=extremely\;\mathrm{poor},\;s_{1}=very\;\mathrm{poor},\;s_{2}=\mathrm{poor},\;s_{3}=slightly\;\mathrm{poor},\;s_{4}=fair,\\ s_{5}=slightly\;\mathrm{good},\;s_{6}=good,s_{7}=very\;\mathrm{good},\;s_{8}=extremely\;\mathrm{good} \end{array}\right\}$$

These are used to compare the four city pairs. Moreover, the weight of each criterion is given by the graduating PhD as *c* = (0.15,0.3,0.4,0.15)^*T*^. Additionally, $\bar{ECI}=0.98$ is set by the graduating PhD.

**Table 2:** The PLPR *H*^1^ based on criteria *c*_1_.

	*A*_1_	*A*_2_	*A*_3_	*A*_4_
*A*_1_	{*s*_4_(1)}	$\left\{s_{3}\left(p_{12,1}^{1}\right),s_{5}\left(p_{12,2}^{1}\right)\right\}$	$\left\{s_{4}\left(p_{13,1}^{1}\right),s_{5}\left(p_{13,2}^{1}\right),s_{6}\left(p_{13,3}^{1}\right)\right\}$	$\left\{s_{4}\left(p_{14,1}^{1}\right),s_{5}\left(p_{14,2}^{1}\right)\right\}$
*A*_2_	/	{*s*_4_(1)}	$\left\{s_{3}\left(p_{23,1}^{1}\right),s_{6}\left(p_{23,2}^{1}\right)\right\}$	$\left\{s_{2}\left(p_{24,1}^{1}\right),s_{4}\left(p_{24,2}^{1}\right)\right\}$
*A*_3_	/	/	{*s*_4_(1)}	$\left\{s_{1}\left(p_{34,1}^{1}\right),s_{4}\left(p_{34,2}^{1}\right)\right\}$
*A*_4_	/	/	/	{*s*_4_(1)}

**Table 3:** The PLPR *H*^2^ based on criteria *c*_2_.

	*A*_1_	*A*_2_	*A*_3_	*A*_4_
*A*_1_	{*s*_4_(1)}	$\left\{s_{3}\left(p_{12,1}^{2}\right),s_{4}\left(p_{12,2}^{2}\right),s_{5}\left(p_{12,3}^{2}\right)\right\}$	$\left\{s_{2}\left(p_{13,1}^{2}\right),s_{3}\left(p_{13,2}^{2}\right)\right\}$	$\left\{s_{5}\left(p_{14,1}^{2}\right),s_{7}\left(p_{14,2}^{2}\right)\right\}$
*A*_2_	/	{*s*_4_(1)}	$\left\{s_{1}\left(p_{23,1}^{2}\right),s_{2}\left(p_{23,2}^{2}\right)\right\}$	$\left\{s_{3}\left(p_{24,1}^{2}\right),s_{5}\left(p_{24,2}^{2}\right)\right\}$
*A*_3_	/	/	{*s*_4_(1)}	$\left\{s_{3}\left(p_{34,1}^{2}\right),s_{6}\left(p_{34,2}^{2}\right)\right\}$
*A*_4_	/	/	/	{*s*_4_(1)}

**Table 4:** The PLPR *H*^3^ based on criteria *c*_3_.

	*A*_1_	*A*_2_	*A*_3_	*A*_4_
*A*_1_	{*s*_4_(1)}	$\left\{s_{5}\left(p_{12,1}^{3}\right),s_{6}\left(p_{12,2}^{3}\right)\right\}$	$\left\{s_{4}\left(p_{13,1}^{3}\right),s_{6}\left(p_{13,2}^{3}\right)\right\}$	{*s*_3_(1)}
*A*_2_	/	{*s*_4_(1)}	$\left\{s_{4}\left(p_{23,1}^{3}\right),s_{6}\left(p_{23,2}^{3}\right)\right\}$	$\left\{s_{4}\left(p_{24,1}^{3}\right),s_{5}\left(p_{24,2}^{3}\right)\right\}$
*A*_3_	/	/	{*s*_4_(1)}	$\left\{s_{3}\left(p_{34,1}^{2}\right),s_{6}\left(p_{34,2}^{2}\right)\right\}$
*A*_4_	/	/	/	{*s*_1_(1)}

**Table 5:** The PLPR *H*^4^ based on criteria *c*_4_.

	*A*_1_	*A*_2_	*A*_3_	*A*_4_
*A*_1_	{*s*_4_(1)}	$\left\{s_{5}\left(p_{12,1}^{4}\right),s_{6}\left(p_{12,2}^{4}\right)\right\}$	$\left\{s_{1}\left(p_{13,1}^{4}\right),s_{5}\left(p_{13,2}^{4}\right)\right\}$	$\left\{s_{6}\left(p_{14,1}^{4}\right),s_{8}\left(p_{14,2}^{4}\right)\right\}$
*A*_2_	/	{*s*_4_(1)}	$\left\{s_{2}\left(p_{23,1}^{4}\right),s_{7}\left(p_{23,2}^{4}\right)\right\}$	$\left\{s_{3}\left(p_{24,1}^{4}\right),s_{5}\left(p_{24,2}^{4}\right)\right\}$
*A*_3_	/	/	{*s*_4_(1)}	{*s*_3_(1)}
*A*_4_	/	/	/	{*s*_4_(1)}

By means of the proposed Eq (14) and the consistency-improving algorithm, we can acquire priority weights of four cities and the occurrence probabilities for four PLPRs as follows:

(1) For the first PLPR, based on criterion *c*_1_, the following mathematical optimization is established based on Eq (14).

$$\begin{array}{l} \min\;f=d_{12}^{+}+d_{12}^{-}+d_{13}^{+}+d_{13}^{-}+d_{14}^{+}+d_{14}^{-}+d_{23}^{+}+d_{23}^{-}+d_{24}^{+}+d_{24}^{-}+d_{34}^{+}+d_{34}^{-}\\ s.t.\left\{ \begin{array}{l} 8\omega_{1}-8\omega_{2}+4-\left(3p_{{}_{12,1}}^{1}+5p_{{}_{12,2}}^{1}\right)-d_{12}^{+}+d_{12}^{-}=0\\ 8\omega_{1}-8\omega_{3}+4-\left(4p_{{}_{13,1}}^{1}+5p_{{}_{13,2}}^{1}+6p_{{}_{13,3}}^{1}\right)-d_{13}^{+}+d_{13}^{-}=0\\ 8\omega_{1}-8\omega_{4}+4-\left(4p_{{}_{14,1}}^{1}+5p_{{}_{14,2}}^{1}\right)-d_{14}^{+}+d_{14}^{-}=0\\ 8\omega_{2}-8\omega_{3}+4-\left(3p_{{}_{23,1}}^{1}+6p_{{}_{23,2}}^{1}\right)-d_{23}^{+}+d_{23}^{-}=0\\ 8\omega_{2}-8\omega_{4}+4-\left(2p_{{}_{24,1}}^{1}+4p_{{}_{24,2}}^{1}\right)-d_{24}^{+}+d_{24}^{-}=0\\ 8\omega_{3}-8\omega_{4}+4-\left(p_{{}_{34,1}}^{1}+4p_{{}_{34,2}}^{1}\right)-d_{34}^{+}+d_{34}^{-}=0\\ p_{{}_{12,1}}^{1}+p_{{}_{12,2}}^{1}=1,p_{{}_{13,1}}^{1}+p_{{}_{13,2}}^{1}+p_{{}_{13,3}}^{1}=1,p_{{}_{14,1}}^{1}+p_{{}_{14,2}}^{1}=1\\ p_{{}_{23,1}}^{1}+p_{{}_{23,2}}^{1}=1,p_{{}_{24,1}}^{1}+p_{{}_{24,2}}^{1}=1,p_{{}_{34,1}}^{1}+p_{{}_{34,2}}^{1}=1\\ \omega_{1}+\omega_{2}+\omega_{3}+\omega_{4}=1\\ p_{{}_{12,1}}^{1},p_{{}_{12,2}}^{1},p_{{}_{13,1}}^{1},p_{{}_{13,2}}^{1},p_{{}_{13,3}}^{1},p_{{}_{14,1}}^{1},p_{{}_{14,2}}^{1},p_{{}_{23,1}}^{1},p_{{}_{23,2}}^{1},p_{{}_{24,1}}^{1},p_{{}_{24,2}}^{1},p_{{}_{34,1}}^{1},p_{{}_{34,2}}^{1}\geq 0\\ d_{12}^{+},d_{12}^{-},d_{13}^{+},d_{13}^{-},d_{14}^{+},d_{14}^{-},d_{23}^{+},d_{23}^{-},d_{24}^{+},d_{24}^{-},d_{34}^{+},d_{34}^{-}\geq 0\\ \omega_{1},\omega_{2},\omega_{3},\omega_{4}\geq 0 \end{array}\right. \end{array}$$

Using MATLAB, we obtain an optimal solution as follows:

$$\begin{array}{l} \omega^{1}={\left(0.3081,0.2311,0.1822,0.2786\right)}^{T},\;p_{{}_{12,1}}^{1}=0.1922,p_{{}_{12,2}}^{1}=0.8078,\;p_{{}_{13,1}}^{1}=0.306,\\ p_{{}_{13,2}}^{1}=0.3812,\;p_{{}_{13,3}}^{1}=0.3128,\;p_{{}_{14,1}}^{1}=0.7644,\;p_{{}_{14,2}}^{1}=0.2356,\;p_{{}_{23,1}}^{1}=0.5362,\;p_{{}_{23,2}}^{1}=0.4638,\\ p_{{}_{24,1}}^{1}=0.19,\;p_{{}_{24,2}}^{1}=0.81,\;p_{{}_{34,1}}^{1}=0.2571,p_{{}_{34,2}}^{1}=0.7429,\;and\\ d_{12}^{+}=d_{12}^{-}=d_{13}^{+}=d_{13}^{-}=d_{14}^{+}=d_{14}^{-}=d_{23}^{+}=d_{23}^{-}=d_{24}^{+}=d_{24}^{-}=d_{34}^{+}=d_{34}^{-}=0. \end{array}$$

Thus, we have $ECI\left(H^{1}\right)=1>\bar{ECI}$, indicating that the consistency level of *H*^1^ achieves satisfaction. Moreover, the complete PLPR, *H*^1^, is obtained by adding the above probabilities of occurrence, as shown in Table 6.

**Table 6:** Four complete PLPR matrices with respect to four criteria.

*H*^1^	*c*_1_	*A*_1_	*A*_2_	*A*_3_	*A*_4_
	*A*_1_	{*s*_4_(1)}	{*s*_3_(0.1922),*s*_5_(0.8078)}	{*s*_4_(0.306),*s*_5_(0.3812),*s*_6_(0.3128)}	{*s*_4_(0.7644),*s*_5_(0.2356)}
	*A*_2_	/	{*s*_4_(1)}	{*s*_3_(0.5362),*s*_6_(0.4638)}	{*s*_2_(0.19),*s*_4_(0.81)}
	*A*_3_	/	/	{*s*_4_(1)}	{*s*_1_(0.2571),*s*_4_(0.7429)}
	*A*_4_	/	/	/	{*s*_4_(1)}
*H*^2^	*c*_2_	*A*_1_	*A*_2_	*A*_3_	*A*_4_
	*A*_1_	{*s*_4_(1)}	{*s*_5_(1)}	{*s*_3_(1)}	{*s*_5_(1)}
	*A*_2_	/	{*s*_4_(1)}	{*s*_2_(1)}	{*s*_3_(0.5),*s*_5_(0.5)}
	*A*_3_	/	/	{*s*_4_(1)}	{*s*_6_(1)}
	*A*_4_	/	/	/	{*s*_4_(1)}
${\tilde{H}}^{3}$	*c*_3_	*A*_1_	*A*_2_	*A*_3_	*A*_4_
	*A*_1_	{*s*_4_(1)}	{*s*_5_(1)}	{*s*_6_(1)}	{*s*_3_(1)}
	*A*_2_	/	{*s*_4_(1)}	{*s*_4_(0.3229),*s*_6_(0.6771)}	{**s**_**2.65**_(**1**)}
	*A*_3_	/	/	{*s*_4_(1)}	{*s*_1_(1)}
	*A*_4_	/	/	/	{*s*_4_(1)}
${\tilde{\tilde{H}}}^{4}$	*c*_4_	*A*_1_	*A*_2_	*A*_3_	*A*_4_
	*A*_1_	{*s*_4_(1)}	{*s*_5_(0.5655),*s*_6_(0.4345)}	{*s*_5_(1)}	{**s**_**5.58**_(**1**)}
	*A*_2_	/	{*s*_4_(1)}	{*s*_2_(0.6314),*s*_7_(0.3686)}	{*s*_3_(0.5619),*s*_5_(0.4381)}
	*A*_3_	/	/	{*s*_4_(1)}	{**s**_**3.76**_(**1**)}
	*A*_4_	/	/	/	{*s*_4_(1)}

(2) Similarly, for the second PLPR, based on criterion *c*_2_, we get
  $$\begin{array}{l} \omega^{2}={\left(0.2812,0.1563,0.4063,0.1563\right)}^{T},\;p_{{}_{12,1}}^{2}=p_{{}_{12,2}}^{2}=0,p_{{}_{12,3}}^{2}=1,\;p_{{}_{13,1}}^{2}=0,p_{{}_{13,2}}^{2}=1,\\ p_{{}_{14,1}}^{2}=1,p_{{}_{14,2}}^{2}=0,p_{{}_{23,1}}^{2}=0,p_{{}_{23,2}}^{2}=1,p_{{}_{24,1}}^{2}=p_{{}_{24,2}}^{2}=0.5,p_{{}_{34,1}}^{2}=0,p_{{}_{34,2}}^{2}=1,\;and\\ d_{12}^{+}=d_{12}^{-}=d_{13}^{+}=d_{13}^{-}=d_{14}^{+}=d_{14}^{-}=d_{23}^{+}=d_{23}^{-}=d_{24}^{+}=d_{24}^{-}=d_{34}^{+}=d_{34}^{-}=0. \end{array}$$
  Thus, we have $ECI\left(H^{2}\right)=1>\bar{ECI}$, which shows that the consistency level of *H*^2^ achieves satisfaction. Moreover, the complete PLPR, *H*^2^, is obtained by adding the above probabilities of occurrence, as shown in Table 6.
(3) For the third PLPR based on criterion *c*_3_, we get
  $$\begin{array}{l} \omega^{3}={\left(0.292,0.2489,0.042,0.417\right)}^{T},\;p_{{}_{12,1}}^{3}=1,\;p_{{}_{12,2}}^{3}=0,p_{{}_{13,1}}^{3}=0,p_{{}_{13,2}}^{3}=1,p_{{}_{23,1}}^{3}=0.1726,\\ p_{{}_{23,2}}^{3}=0.8274,\;p_{{}_{24,1}}^{3}=1,p_{{}_{24,2}}^{3}=0,\;and\;d_{12}^{-}=0.6548,d_{24}^{-}=1.3452,\\ d_{12}^{+}=d_{13}^{+}=d_{13}^{-}=d_{14}^{+}=d_{14}^{-}=d_{23}^{+}=d_{23}^{-}=d_{24}^{+}=d_{34}^{+}=d_{34}^{-}=0. \end{array}$$
  Thus, we have *ECI*(*H*^3^) = 0.9583<0.98, indicating that the consistency level of *H*^3^ does not achieve satisfaction. Therefore, the consistency-improving algorithm must be utilized to obtain an adjusted *H*^3^ as follows:
  According to Eq (16), we have $d^{\max}=d_{24}^{-}=1.3452$. Then, according to ${\tilde{L}}_{24,k}=I^{-1}\left(I\left(L_{24,k}\right)-d_{24}^{-}\right)\left(k=1,2\right)$, we obtain ${\tilde{L}}_{24,1}=s_{2.65}$ and ${\tilde{L}}_{24,2}=s_{3.65}$. Then, the corrective PLTS is ${\tilde{L}}_{24}=\left\{s_{2.65}\left(p_{24,1}\right),s_{3.65}\left(p_{24,2}\right)\right\}$. Thus, another corrective PLTS is ${\tilde{L}}_{42}=\left\{s_{5.35}\left(p_{42,1}\right),s_{4.35}\left(p_{42,2}\right)\right\}$. Thus, the corrective PLPR can be constructed by introducing ${\tilde{L}}_{24}$ and ${\tilde{L}}_{42}$.
  Using Eq (14) again, we obtain the optimal solution as follows:
  $$\begin{array}{l} {\bar{\omega }}^{3}={\left(0.3014,0.2207,0.0514,0.4264\right)}^{T},\;p_{{}_{12,1}}^{3}=1,p_{{}_{12,2}}^{3}=0,\;p_{{}_{13,1}}^{3}=0,p_{{}_{13,2}}^{3}=1,\\ p_{{}_{23,1}}^{3}=0.3229,p_{{}_{23,2}}^{3}=0.6771,\;p_{{}_{24,1}}^{3}=1,p_{{}_{24,2}}^{3}=0,\;d_{12}^{-}=0.3543,d_{24}^{-}=2957,\\ d_{12}^{+}=d_{13}^{+}=d_{13}^{-}=d_{14}^{+}=d_{14}^{-}=d_{23}^{+}=d_{23}^{-}=d_{24}^{+}=d_{34}^{+}=d_{34}^{-}=0. \end{array}$$
  Thus, we get $ECI\left({\tilde{H}}^{3}\right)=0.9865>0.98$, showing that ${\tilde{H}}^{3}$ achieves the acceptable consistency level. Moreover, the complete PLPR, ${\tilde{H}}^{3}$, is obtained by adding the above probabilities of occurrence, as shown in Table 6.
(4) For the fourth PLPR, based on criterion *c*_4_, we obtain the optimal solution as follows:
  $$\begin{array}{l} \omega^{4}={\left(0.385,0.2045,0.1902,0.2203\right)}^{T},\;p_{{}_{12,1}}^{4}=0.5567,p_{{}_{12,2}}^{4}=0.4433,\;p_{{}_{13,1}}^{4}=0,p_{{}_{13,2}}^{4}=1,\\ p_{{}_{14,1}}^{4}=1,\;p_{{}_{14,2}}^{4}=0,\;p_{{}_{23,1}}^{4}=0.577,p_{{}_{23,2}}^{4}=0.423,\;p_{{}_{24,1}}^{4}=0.5631,p_{{}_{24,2}}^{4}=0.4369,\\ d_{13}^{+}=0.5583,d_{14}^{-}=0.683,d_{34}^{+}=0.7588\\ d_{12}^{+}=d_{12}^{-}=d_{13}^{-}=d_{14}^{+}=d_{23}^{+}=d_{23}^{-}=d_{24}^{+}=d_{24}^{-}=d_{34}^{-}=0. \end{array}$$
  Thus, we obtain *ECI*(*H*^4^) = 0.9583<0.98, showing that the consistency level of *H*^4^ is dissatisfactory. Therefore, the consistency-improving algorithm is used to improve the consistency level of *H*^4^. After two iterative-improving algorithms, the complete PLPR, ${\tilde{\tilde{H}}}^{4}$, achieves an acceptable consistency level. The improving processes are omitted. Finally, we obtain the following optimal solutions by means of Eq (14)
  $$\begin{array}{l} {\bar{\bar{\omega }}}^{4}={\left(0.3757,0.1964,0.216,0.2119\right)}^{T};\;p_{{}_{12,1}}^{4}=0.5655,p_{{}_{12,2}}^{4}=0.4345,\;p_{{}_{13,1}}^{4}=0,p_{{}_{13,2}}^{4}=1,\\ p_{{}_{14,1}}^{4}=1,\;p_{{}_{14,2}}^{4}=0,\;p_{{}_{23,1}}^{4}=0.6314,p_{{}_{23,2}}^{4}=0.3686,\;p_{{}_{24,1}}^{4}=0.5619,p_{{}_{24,2}}^{4}=0.4381;\\ d_{13}^{+}=0.2775,d_{14}^{-}=0.2692,d_{34}^{+}=0.2733,\\ d_{12}^{+}=d_{12}^{-}=d_{13}^{-}=d_{14}^{+}=d_{23}^{+}=d_{23}^{-}=d_{24}^{+}=d_{24}^{-}=d_{34}^{-}=0. \end{array}$$
  Thus, we obtain $ECI\left({\tilde{\tilde{H}}}^{4}\right)=0.9829>0.98$, indicating that the consistency level of ${\tilde{\tilde{H}}}^{4}$ achieves an acceptable level. Furthermore, the complete PLPR, ${\tilde{\tilde{H}}}^{4}$, is obtained by adding the above probabilities of occurrence, as shown in Table 6.

Finally, the ranking matrix of four cities is obtained as follows:

$$\begin{array}{l} \begin{array}{cccc} \;c_{1} & \;c_{2} & \;c_{3} & \;c_{4} \end{array}\\ \left[\begin{array}{cccc} 0.3081 & 0.2812 & 0.3014 & 0.3757\\ 0.2311 & 0.1563 & 0.2207 & 0.1964\\ 0.1822 & 0.4063 & 0.0514 & 0.216\\ 0.2786 & 0.1563 & 0.4264 & 0.2119 \end{array}\right]\times \left[\begin{array}{c} 0.15\\ 0.3\\ 0.4\\ 0.15 \end{array}\right]=\left[\begin{array}{c} 0.3075\\ 0.1993\\ 0.2022\\ 0.2910 \end{array}\right]\begin{array}{c} \leftarrow x_{1}\\ \leftarrow x_{2}\\ \leftarrow x_{3}\\ \leftarrow x_{4} \end{array} \end{array}.$$

Therefore, the ranking of four cities is *x*_1_≻*x*_4_≻*x*_3_≻*x*_2_. *x*_1_ (Qing Dao) is the best selection for the graduating PhD.

### Contrastive analysis

We have defined the consistency level and the acceptable consistency of PLPR. For a PLPR with an unacceptable consistency, an iteration algorithm was designed to improve its consistency until it was acceptable. As compared to the method of Zhang et al. [19], we summarized the advantages of our method as follows:

1. Our method can be applied to derive the priority weights of PLPR with unknown probabilities, whereas the Zhang et al. [19] method cannot.
2. The consistency-improvement processes of the two methods differ. Zhang et al. [19] adjusted all elements each time to improve the consistency level of PLPR, whereas our method only adjusted the most inconsistent elements each time, based on the probability computing model. This should preserve the initial information of DMs as far as possible.
3. Our method can directly obtain the priority weights of alternatives with the probability computing model, whereas Zhang et al. [19] carries out a normalization process and a PLWA operator integrating process. Therefore, our method greatly reduced the computational burden.

## Conclusions

PLTS comprises HFLTSs and their corresponding occurrence probabilities, which contain more precise information than HFLTSs. However, it is either impossible or against their will for DMs to provide precise probability values for all linguistic terms in the PLPR. Therefore, based on the defined expected consistency, a probability computing model was developed to calculate the probability values of the PLPR. Thus, a complete PLPR could be obtained. During real decision-making, the PLPR may be inconsistent because of the limitation of DM knowledge. Based on the defined ECI of the PLPR, a consistency-improving iterative algorithm was established to improve the consistency level by adjusting certain elements of PLPR. When the ECI of PLPR achieves or exceeds the default satisfactory level, the consistency-improving algorithm is finished. This consistency-improving and reaching process ensures that DMs’ preference information is logical. By using a probability computing model and a consistency-improving algorithm with PLPR, the optimal alternative could be obtained. Finally, by solving the employment-city selection problem, the feasibility and effectiveness of our methods were further validated from the application view.

In the future, the proposed methods should be applied to the practical large-scale group decision-making problems. Moreover, a consensus-reaching model, based on PLPRs, is also an interesting research direction.
